# Tachdjian's Pediatric Orthopaedics: from the Texas Scottish Rite Hospital for Children

**DOI:** 10.5704/MOJ.1503.012

**Published:** 2015-03

**Authors:** Sharaf Ibrahim

**Affiliations:** Department of Orthopaedics and Traumatology Faculty of Medicine, Universiti Kebangsaan Malaysia

Dr herring and his colleagues at the Texas Scottish Rite Hospital for Children have produced the latest edition of Tachdjian's Pediatric Orthopaedics combining a hard copy with an online version

This is a two-volume book with the complete three-volume digital version available online. The online access via expertconsult.inkling.com requires a user name and password. The hard copy has 34 chapters. Chapters 35 to 43 are online and comprise neuromuscular and other orthopaedic disorders. Fifty-eight videos of operative procedures, scoliosis casting techniques and patients' clinical examination are available online.

The videos include most of the major surgical procedures in paediatric orthopaedics such as the growing rod for scoliosis, open reduction for developmental dysplasia of the hip, pelvic osteotomies, surgical hip dislocation for slipped capital femoral epiphysis, triple arthrodesis of the foot and the 8-plate.

The quality of some of the videos however, could be improved with further editing as the operative field was sometimes obstructed by the surgeon or too small to visualize.

The videos on physical examinations show the clinical features of a child with asymmetric abdominal reflexes, an infant with femoral nerve palsy after using a Pavlik Harness and children with unilateral and bilateral developmental dysplasia of the hips.

There are numerous computer-aided colour illustrations throughout the book including 44 operative procedures in the hard copy and another 33 procedures online. The illustrations provide excellent educational and aesthetic value to the book.

The book does not have a CD-ROM and accessing the book online will need an internet connection.

Overall, this is an excellent textbook on paediatric orthopaedics.

